# Male Partners Involvement in Prevention of Mother-to-Child Transmission of HIV Services in Southern Central Ethiopia: In Case of Lemo District, Hadiya Zone

**DOI:** 10.1155/2017/8617540

**Published:** 2017-03-15

**Authors:** Degefa Tadele Belato, Abera Beyamo Mekiso, Bayu Begashaw

**Affiliations:** ^1^Hadiya Zone Health Department, Hadiya Zone, Ethiopia; ^2^Public Health Department, College of Health Sciences, Wolayta Sodo University, Wolayta Sodo, Ethiopia; ^3^Public Health Department, College of Health Sciences, Mizan-Tepi University, Mizan-Aman, Ethiopia; ^4^College of Medicine and Health Sciences, Institute of Public Health, University of Gondar, Gondar, Ethiopia

## Abstract

Male partners' involvement is a vital issue to prevent human immunodeficiency virus (HIV) transmission from mother to child; because it is much expectable that women were more vulnerable and high risk group of population portion. Therefore, to save lives of mothers and their newborn from acquiring HIV, male partners should do their maximum endeavor regardless of any determinant factors as our results revealed its status in our study context and elsewhere at past time too.

## 1. Background

Mother-to-child transmission is the most common mode of human immunodeficiency virus (HIV) transmission in children which can be vertically transmitted from HIV positive pregnant women to their unborn babies during pregnancy, labour, and delivery or through breastfeeding after delivery [1–3]. HIV remains a major challenge globally regardless of decades of advocacy and investment in programs to control the spread of the virus [4, 5]. Globally, an estimated 35.3 million people were living with HIV in 2012 [6, 7]. The number of newly infected children in 2012 was 260,000 in low- and middle-income countries [7]. Nearly all of these children acquired HIV through mother-to-child transmission and ninety percent of them live in Sub-Saharan Africa [8, 9]. Beyond this HIV/AIDS mainly affects people of reproductive age and increasingly affects women whose newborn babies will be victims of the disease [9]. Consequently, maximum efforts to improve programs for prevention of mother-to-child transmission (PMTCT) of HIV have been focused primarily at the health facility level on defining an effective set of community interventions to increase demand and uptake of services and retention [10]. Among communities male partners have greater roles but their participation is low in PMTCT [11].

Uptake of PMTCT services by the pregnant mothers is affected both by factors related to the health system such as accessibility of voluntary counselling and testing (VCT) services and by individual factors such as fear of disclosure of HIV results, lack of male partner support, fear of domestic violence, rejection, and negligence [1, 5, 11–14]. In Ethiopia, mother-to-child transmission (MTCT) accounts for 95% of childhood HIV infections. It poses a significant threat to child health and survival [11, 13, 15]. In 2013, there were an estimated 793,700 people living with HIV including 200,300 children and the paediatric HIV population. In the country there were mostly older children who were vertically infected in earlier years when the coverage and effectiveness of PMTCT in the country were low [11]. To halt the problem their male partners and mostly husbands should play a vital role besides mothers [16].

In Ethiopia, lack of male partner involvement in PMTCT undermined the potential benefits of antenatal HIV preventive efforts [16], thus representing a missed opportunity to effectively prevent vertical HIV transmission [17]. However, the factors associated with poor male participation are not well known in Ethiopia and need to be investigated to help the country develop reasonable strategies to address these bottlenecks. Male involvement is said to be very low in many health facilities in Ethiopia and it is one of the potential program gaps unfavourably affecting PMTCT services uptake in the country [18]. For this reason, previously in a country it was highlighted as involving male partners in ANC/PMTCT can often be utilized as an entry point for the provision of additional PMTCT services notably partner testing, condom use, and infant feeding recommendations for both male and female participants [6, 14].

There is global consensus that the world must strive towards elimination of new HIV infections among children by 2015 to keep mothers and children living with HIV alive. Many low- and middle-income countries have already moved significantly towards achieving these goals [19]. In spite of this in Southern Ethiopia, only 45% mothers brought their partner to HIV counselling and testing. Among these only 25% were tested and the HIV prevalence was 6.5%. This can also increase the likelihood of HIV infection among the newborns [17, 20].

As a result, in Lemo district, there is no clear evidence that could show level of male partners involvement in the PMTCT service. Thus, this study was conducted to determine the level of male involvement in PMTCT and factors which affect male involvement in PMTCT of HIV among males whose wives were attending ANC in previous six months before the study was conducted in Lemo district, Hadiya Zone, southern central Ethiopia.

## 2. Materials and Methods

### 2.1. Study Design, Setting, and Participants

Community based cross-sectional study was conducted in Lemo district from March 15/2015 to April 15/2015. Lemo district is located about 213 km from Addis Ababa, the capital city of Ethiopia. Based on 2007 national census, projection for 2014/15 the total population of the District is 148,339. From this 73,390 were males and 74,950 were females. There were 34,563 women in reproductive age group from which 5133 is expected pregnancy and 4732 were under one-year children [21].

### 2.2. Sampling

The sample size was calculated using a single population proportion formula, considering 50% proportion of male partner involvement in PMTCT of HIV service, 5% margin of error, design effect 2, correction formula, and 10% estimated nonresponse rate.

Multistage stratified sampling technique was used to select the study units. First, all the* kebeles* (the smallest administrative unit in Ethiopian government system below a district) in the district was stratified into urban and rural kebeles. Then 1 out of 2 urban kebeles and 10 out of 33 rural kebeles were selected randomly. The number of male partners was proportionally allocated based on the average number of male partners whose pregnant mothers were attending ANC services in previous six months before the study was conducted. Family folder in the health posts was used to identify households with eligible male partners. Households with eligible male partners were identified and sampling frame was created based on house numbers. Finally computer generated simple random sampling method was employed to select and approach each male partner.

### 2.3. Variables in the Study and Its Measurement

Data was collected by using interviewer administered structured questionnaire that was initially adapted from previous studies in English (questionnaire, in Supplementary Material available online at https://doi.org/10.1155/2017/8617540) and then was translated to both Hadiyigna and Amharic by language experts in Hadiyigna and Amharic and translated back to English by another language expert to ensure consistency. The tool includes five sections; sociodemographic and socioeconomic factors of the respondents, sociocultural norms of the respondents, service related factors of PMTCT, knowledge of respondents in PMTCT of HIV, and level of male involvement in PMTCT services. Those who responded to five and above male involvement items were recorded as “yes = 1” and those who responded to less than five items were recorded as “no = 0”; respondents' [11] total score on male involvement was calculated by summing up the scores of ten items designed to assess level of male involvement. All items had an equal weight of score 1. For all items, a score of 1 was given for “yes” responses for positive connotation and 0 (zero) for “no” or “uncertain” responses for negative connotation.

### 2.4. Data Collection and Processing

Eight diploma nurses for data collection and two health officers for supervision were recruited. One day training on how to fill the questionnaire, request the consent, and assure confidentiality of information of the study participants was given to data collectors and supervisors to ensure the quality of the field operation by principal investigator. During data collection, the supervisors had supervised the data collection process in daily base and performed quality checks. The instrument was pretested on 5% of the sample in Misha District which is 18 km far from the study area to check reliability of the tool. The pretest data was not included in the main data analysis.

All collected data from questionnaire was checked manually before entry to software. Then, the data was entered into a computer using EpiData version 3.1 software. The software was created based on data type and size, categories, validating permitted values and ranges, and codes to missing value. Corrections were made according to the original data.

### 2.5. Data Analysis

The data was analyzed by using SPSS version 16 software. Descriptive analysis was carried out for each of the variables to check frequency, distribution, and missing value. Bivariate analysis was employed to check crude association between level of male partner involvement in PMTCT of HIV services and independent variables. Chi-square test was conducted to see variables fulfilling assumption. Variable with *p* value < 0.25 on bivariate analysis was entered to multivariable logistic regression to identify the factors that affect male involvement in PMTCT. Binary logistic regression test was used to assess association between independent variables and male involvement in PMTCT. Odds ratio and corresponding 95% confidence intervals were used to quantify the degrees of association between independent variable and level of male partner involvement in PMTCT of HIV services. Results with *p* value ≤ 0.05 were considered as being statistically significant and the rest was refuted. Multicollinearity among independently associated variables was checked by multicollinearity diagnostic test VIF in linear regression and none was collinear.

### 2.6. Ethical Statement

Ethical clearance and formal letters were obtained from Hadiya Zonal Health Department; then letter of permission was produced from administrative bodies of the zone to the district administration hierarchically. Verbal consent was preferred rather than written consent for the following reasons. Firstly, this was cross-sectional study that found out descriptive data. Secondly, their responses had no personal, cultural, social, or political impacts. Thirdly, there would not be significant risk/s to the participants. Finally, a significant number of people living in the study area have no educational status. The IRB approved the proposed verbal consent procedure. The confidentiality of the data was ensured by not asking their names.

## 3. Results

### 3.1. Study Participant Characteristics

Total of 401 male partners of pregnant mothers had participated in thestudy making the response rate of 95%. The remaining 5% were not taking part during interview due to lack of consent, withdraw, and fear of social neglect. The majority, 173 (43.1%), of respondents belong to the age group below 34 years with median age of 35 years. Regarding the ethnic background the predominant ethnic group, 341 (85%), was Hadiya followed by Kambata, 30 (7.5), while the dominant religion, 242 (60.2%), was protestant followed by orthodox 85 (21.2%). The majority, 378 (94.4%), of male partners were from the rural areas; the remaining 23 (5.7%) of respondents were from the urban areas. With regard to occupation, 176 (68.8%) of the participants were farmers followed by merchants 75 (18.7%) and the largest number of the respondents, 297 (74.1%), had earned family monthly income above 1170 ETB (Table 1).

### 3.2. Sociocultural Factors Influence on Male Involvement in PMTCT of HIV Services

Only 123/401 (30.9%) were accompanied by their male partner at the ANC/PMTCT. Among male partners who accompany their pregnant wives, 117/123 (95.2%) of husbands who accompany their pregnant wives have tested and counselled for HIV in the current pregnancy of their wives at ANC. Three-fourth of the respondents (64.1%) agreed that it was taboo for men to discuss with women HIV testing during pregnancy, delivery, and breastfeeding. Nearly half of the respondents, 183 (45.6%), agreed that it is better to postpone HIV testing to postdelivery due to fear of stress during pregnancy and 252 (62.8%) of respondent approved that it is enough that a pregnant woman come to ANC clinic by less busy relatives or friends. Nearly half of respondents, 196 (48.8%), agreed that the maternal and child health clinics should focus only on the health care service of women and children excluding male partners and 174 (43.4%) of the respondents decided that if the pregnant woman was found to be HIV positive she should be divorced.

### 3.3. Male Partners Knowledge in PMTCT of HIV Services

Three hundred forty-seven (86.5%) of male partners knew that HIV could be transmitted by an unprotected sexual intercourse. Almost half, (50.4%) and (50.6%), of male partners knew that MTCT of HIV could be during pregnancy and labour, respectively, and 169 (42.1%) of respondents approved that HIV could be transmitted during breastfeeding. Nearly half, 182 (45%), of male partners knew that HIV counselling and testing of pregnant women could reduce MTCT of HIV and almost one-fourth (27.4%) of male partners knew that HIV counselling and testing of pregnant women could reduce MTCT of HIV and nearly three-fourth (72.6%) of the male partners knew that provision of ARV could reduce MTCT of HIV. About half (49.6%) of male partners knew and heard about PMTCT services and almost half (51.9%) of male partners knew that HIV counselling and testing service was provided for pregnant women in ANC clinics.

### 3.4. Service Related Factors to Male Partner Involvement in PMTCT of HIV

Two hundred nineteen (54.6%) of male partners disproved that antenatal clinics should be opened on weekends and evening for men to attend ANC clinics with their partner and 72 (17.9%) of respondents agreed that distance from health facility was major obstacle to have attend ANC/PMTCT clinic with their partners and also 214 (53.4%) of male partners disagreed that couple HIV counselling and testing for PMTCT should be conducted at villages.

Majority, 249 (62.0%), of the respondents agreed that there should be separate waiting areas for men and women visiting maternal and child health clinics and 214 (53.4%) of male partners approved that there should be a different exit for male and female partners after HIV testing to avoid being identified by the crowded waiting for service. Nearly two-thirds of the respondents (66.0%) agreed that service providers did not request men to enter into ANC room together with their partner and nearly one-fifth and 79 (19.7%) of male partners agreed that health facilities did give men medical certificate of ANC attendance and invited verbally or in written form for their attendance at ANC clinic by the antenatal clinic, respectively.

### 3.5. Level of Male Partner's Involvement in PMTCT of HIV Services

Nearly two-thirds, 124 (30.9), of male partners supported ANC follow-up of their partners by arranging transport cost, 122 (30.4%) of male partners were self-initiated to discuss HIV testing with their partner during pregnancy, 125 (31.2%) of male partners participated by remembering the follow-up schedule, and 117 (29.2%) of male partners were accompanying the partner to ANC clinics at least once; out of those who came to ANC clinic with their partners only 61 (15.2%) of male partners entered into ANC room together with their partner. Only one hundred eleven (28.0%) male partners were counselled and tested for HIV during their partners' pregnancy; out of those counselled and tested only 45 (38.9%) of male partners were counselled and tested together with their female partner.

Almost three-fourth of the respondents (76.8%) agreed to support the medical follow-up of newborn until their HIV status was known while nearly two-thirds of the respondents, 259 (64.6%), were confident of using a condom consistently in case of discordant HIV status but nearly one-fourth of the male partners (24.4%) decided to discontinue conjugal or love relationship if their partners' HIV status was positive.

### 3.6. Factors Affecting Male Partners Involvement in PMTCT of HIV Service

The bivariate analysis revealed that residence, level of education, occupation and family monthly income, sociocultural factors influence, level of knowledge, and service related factors influences were candidates for further analysis in multivariable logistic regression. Variables such as level of education, occupation, family monthly income, sociocultural factors influence, level of knowledge, and service related factors influences were significantly associated with male partners involvement in PMTCT of HIV service.

### 3.7. Factors Independently Affecting Male Partners Involvement in PMTCT Services

Variables which significantly predict the level of male partners' involvement in PMTCT of HIV services were family monthly income, level of education, sociocultural factor influence, level of knowledge, and service related factors influence. See Table 2.

Those male partners educated from grades 1 to 4 were (AOR, 0.27, 95% CI: 0.08–0.90) less likely involved in PMTCT of HIV services when compared with grade 11 and higher. Male partners who had family income ≤ 1170 ETB monthly were 84% (AOR, 0.16, 95% CI: 0.1–0.4) less likely involved in PMTCT service when compared to those who had earned 1170 ETB. Male partners who had low sociocultural factor influence to involve in PMTCT services were almost four (AOR = 4.09, 95% CI: 2.28–7.34) times more likely involved in PMTCT services when compared with their counterparts. Moreover, the participants who had high knowledge in PMTCT of HIV service were nearly seven (AOR = 6.65, 95%: 3.67–12.08) times more likely involved in PMTCT service when compared with their counterparts and the respondents who had better service related factors influence to involve in PMTCT service were almost three (AOR, 3.2, 95% CI: 1.78–5.63) times more likely involved in PMTCT services when compared with those who had high service related factor influence to be involved in PMTCT of HIV services.

## 4. Discussion

In this study the level of male partner involvement in prevention of mother-to-child transmission of human immune deficiency virus service among participated male partners was 30.9%. The findings of this study were higher than the studies result which were conducted in Gondar and Mekelle which revealed that 20.9% and 20.1% of male partners come to ANC/PMTCT clinic with their female partners, respectively [11, 20]. The discrepancy of these findings might be attributed to difference in method used and study settings, sociodemographic characteristics of the study participants, and availability and accessibility of the infrastructures.

In this study, 15.2% of male partners who had come to ANC/PMTCT clinic with their partners were entered into ANC room together with their partners. This finding is lower than that of the study conducted in Deremarkos town which revealed that out of respondents who had come with their partners to ANC clinic, 69.3% of male partners were entered into ANC room together with their female partners [22].

One hundred twenty-three (30.7%) of male partners were highly involved in the PMTCT of HIV services in this study. This finding is nearly similar with the study conducted in four districts in Addis Ababa which found that 28.1% of male partners were highly involved in PMTCT of HIV services and the study in Eastern Uganda found that 99 (26%) of the male partners were highly involved in PMTCT services. However, the finding of this study is lower than the study conducted in Debre Markos town and three public hospitals in Addis Ababa which revealed that male partner involvement in the PMTCT services was 72.26% and 88, respectively. This difference might be due to the fact that those studies were conducted in urban area, with probable higher access to information on PMTCT of HIV services. This implies that efforts should be made to disseminate information through different mechanisms to community about PMTCT of HIV services in the study area.

Male partners educated from grades 1 to 4 were 73% less likely involved in PMTCT services when compared with grades 5 to 8, whereas, male partners educated till grade 11 and higher were almost three times more likely involved in PMTC services when compared with grades 5 to 8 in this study. The findings of this study are supported by the study findings conducted in Eastern Uganda which found that male partners who had attended secondary education or higher education were twice more likely involved in PMTCT of HIV services. The findings of this study imply that formal education has impact on male partners involvement in PMTCT services; therefore, more efforte should be made to increase awareness to low educated individuals about PMTCT services to increase PMTCT uptake in the study area.

This study found that male partners who had earned average family monthly income less than or equal 1170 ETB were 84% less likely involved in PMTCT of HIV service when compared to those who earned monthly income above 1170 ETB. The findings of this study supported the study conducted in South Africa which revealed that the male partners who had high monthly income were more involved in PMTCT of HIV services than respondents who had low monthly income [1]. This low involvement might be due to lack of awareness about PMTCT service which are given free of fee in all governmental health facilities and sociodemographic characteristics of the respondents. This implies that more efforts should be made to create awareness to community as PMTCT service is given free of fee for both pregnant mothers and their partners found in any economical status to increase level of male partners involvement in PMTCT services in the study area.

This study found that 180 (45.2%) of male partners had low sociocultural factors influence to be involved in PMTCT services. The findings of this study are inconsistence with the study finding in Debre Markos town which revealed 99.3% of male partners were within the range of low sociocultural factors influence [22]. This discrepancy might be attributed to different sociodemographic characteristics of respondents; in the later study more than two-thirds of the respondents were farmers whereas in previous study more than half of the respondents were government employees and study context also has its own impact for the observed discrepancy.

In the current study male partners who had low sociocultural factors influence on being involved in PMTCT services were almost four times more likely to be involved in PMTCT of HIV service when compared to male partners who had high sociocultural factors influence on being involved in PMTCT services. The finding of this study was comparable with the findings of studies conducted in Addis Ababa which revealed that as the sociocultural influence decreases the males involvement in PMTCT increases [23]. The findings of this study are also supported by the study findings in South Africa, which state that the barriers to male partner testing during pregnancy, delivery, and breast feeding were fear, guilt, and subsequent denial associated with a positive test result and lack of social expectation for a man to get tested during his partner's pregnancy [24].

This study found that 46.1% of male partners had high knowledge in PMTCT of HIV services. The findings of this study are lower than the study finding from four districts of Addis Ababa which revealed that 77% of male partners had high knowledge in PMTCT of HIV services [23]. The discrepancy might be attributed to difference in sociodemographic characteristics of the study participants and lack of access and availability of infrastructures like mass media and others.

Moreover, this study revealed that male partners who had high knowledge in PMTCT of HIV service were nearly seven times more likely involved in PMTCT of HIV services when compared to those who had low knowledge in PMTCT services. The findings of this study are comparable to the study findings from four districts of Addis Ababa which revealed that participants who had high knowledge were 14% more likely to have high involvement in PMTCT services when compared to those who had low knowledge [23]. Furthermore, the findings of this study are supported by the study findings in Debre Markos town which revealed that male partners who had moderate and good knowledge about PMTCT services were 4.4 and 3.2 times more likely involved in PMTCT services than male partners with low knowledge about PMTCT services, respectively [22].

Male partners having better service related factor influence to be involved in PMTCT services were 44.9%. This finding is lower than study conducted in Nigeria which revealed that 82.4% of male partners had low service related factor influence to involve in PMTCT services [5].

Moreover, respondents who had better service related factors influence on PMTCT service were almost three times more likely involved in PMTCT services when compared to their counterparts. The findings of the study are consistent with the study findings from Debre Markos town which showed that the respondents who had low service related factor influence to involve in PMTCT service were 14 times more likely involved in PMTCT of HIV services [24]. Lastly, our study revealed that, among male partners who accompany their pregnant wives, 117 (95.2%) of husbands who accompany their pregnant wives have tested and counselled for HIV in the current pregnancy of their wives at ANC. This is higher than findings from Mekele and Tanzania [11, 25]. This inconsistency might be due to current improvement in maternal and child health activities when compared to those studies period.

## 5. Conclusions

In this study majority of male partners had low involvement in PMTCT services. Male partners who are highly educated and have high family monthly income, high knowledge, low sociocultural influence toward male partners' involvement in PMTCT services, and low service related factor influence toward male partner involvement in PMTCT services were more likely involved in PMTCT of HIV services than their counterparts. But lack of awareness and knowledge about PMTCT services, cultural influence, and service related factors influence were some of the reasons that hinder higher male partner involvement in PMTCT of HIV services. Low male involvement in PMTCT service affects successful implementation of the PMTCT program. Additional consideration should also be given for the risk factors which may increase maternal and child mortality due to high expansion of HIV from mother-to-child. Availability of PMTCT service should be disseminated to community in different media with local language to increase the knowledge of male partners and to decrease sociocultural factors influence in PMTCT service. We also recommend further countrywide research on the issue.

## Supplementary Material

The questionnaire includes overall questions regarding male partners involvement, experience and opinion on PMTCT.

## Figures and Tables

**Table 1:** Showing sociodemographic and economic characteristic of male partners' involvement in PMTCT of HIV services in Lemo District, Hadiya Zone, southern central Ethiopia, 2015.

Sociodemographic variables	Frequency (*n* = 401)	Percent (%)
Residence		
Urban	23	5.7
Rural	378	94.3
Age		
≤34 years	173	43.1
35–44 years	157	39.2
≥45 years	71	17.7
Marital status		
Traditional married	371	92.5
Registered married	30	7.5
Duration of living relationship		
<5 years	143	35.7
5–10 years	132	32.9
>10 years	126	31.4
Religion		
Protestant	242	60.3
Orthodox	85	21.2
Muslim	54	13.5
Others^a^	20	5.0
Ethnicity		
Hadiya	341	85.0
Kambata	30	7.5
Silte	16	4.0
Others^b^	14	3.5
Educational		
Illiterate	18	4.5
Able to read and write	26	6.5
Grades 1–4	58	14.5
Grades 5–8	134	33.4
Grades 9-10	84	20.9
Grade 11 and higher	81	20.2
Occupation		
Farmers	276	68.8
Governmental employees	36	9.0
Merchants	75	18.7
Daily labourers	14	3.5
Monthly income		
≤1170 ETB	104	25.9
>1170 ETB	297	74.1

^a^Catholic and Adventist ^b^Wolayta and Halaba.

**Table 2:** Showing bivariate and multivariable logistic regression of factors associated with male partners' involvement in PMTCT of HIV among males in Lemo district, Hadiya Zone, southern central Ethiopia, 2015.

Variables	Male partner involvement in PMTCT of HIV services				
	High	Low	COR (95% C.I)	AOR (95% C.I)	*p* value
	Number (%)	Number (%)			
Residence					
Urban	10 (43.5)	13 (56.5)	1.81 (0.77, 4.24)	3.08 (0.91, 10.42)	0.071
Rural	113 (29.9)	265 (70.1)		1	
Educational status					
Illiterate	5 (27.8)	13 (72.2)	1.28 (0.42, 3.86)	0.72 (0.19, 2.64)	0.619
Able to read and write	6 (23.1)	20 (76.9)	0.99 (0.37, 2.70)	0.49 (0.14, 1.74)	0.276
Grades 1–4	5 (8.6)	53 (91.4)	0.31 (0.12, 0.85)	0.27 (0.08, 0.90)	0. 033^*∗*^
Grades 5–8	31 (23.1)	103 (76.9)	1.41 (0.76, 2.61)	0.76 (0.35, 1.64)	0.22
Grades 9-10	25 (29.8)	59 (70.2)	5.61 (3.09, 10.33)	3.19 (0.54, 6.64)	0.488
Grade 11 and higher	51 (63)	30 (30.0)		1	
Occupation					
Farmer	62 (22.5)	214 (77.5)		1	
Governmental employee	30 (83.3)	6 (16.7)	17.26 (6.87, 43.35)	1.75 (0.47, 6.47)	0.403
Merchant	28 (37.3)	47 (62.7)	2.06 (1.19, 3.55)	0.67 (0.31, 1.46)	0.413
Daily laborer	3 (21.7)	11 (78.9)	0.94 (0.26, 3.48)	1.11 (0.17, 7.39)	0.915
Monthly income					
≤1170 ETB	11 (10.6)	93 (89.4)	0.19 (0.1, 0.38)	0.16 (0.1, 0.4)	0.000^*∗*^
>1170 ETB	112 (37.7)	185 (62.3)		1	
Sociocultural factors					
High cultural	42 (10.6)	176 (44.2)		1	
Low cultural	80 (20.1)	100 (25.1)	3.35 (2.14, 5.24)	4.09 (2.28, 7.34)	0.000^*∗*^
Level of knowledge					
High knowledge	96 (30.7)	89 (69.3)	7.55 (4.60, 12.40)	6.65 (3.67, 12.08)	0.000^*∗*^
Low knowledge	27 (6.7)	189 (47.1)		1	
Service related factors					
Poor service	44 (11.0)	176 (44.1)		1	
Better service	78 (19.5)	101 (25.3)	3.09 (1.98, 4.81)	3.17 (1.78, 5.63)	0.000^*∗*^

*Note*. ^*∗*^= statistically significant at (*p* ≤ 0.05); 1 = reference group.

## Abbreviations

ANC: Antenatal care
ETB: Ethiopian birr
HIV: Human immune deficiency syndrome
MTCT: Mother-to-child transmission
PMTCT: Prevention of mother-to-child transmission
SNNPR: Southern nation nationalities and peoples region.

## Data Access

All relevant data are within the paper and its supporting information files but any additional data required by the journal can be available anytime.
