# Supplementary material for: Male Partners Involvement in Prevention of Mother-to-Child Transmission of HIV Services in Southern Central Ethiopia: In Case of Lemo District, Hadiya Zone
Source: AIDS Res Treat. 2017 Mar 15;2017:8617540. doi: 10.1155/2017/8617540 (PMC5376926; doi:10.1155/2017/8617540)
Supplement: Supplementary file 1 — The questionnaire includes overall questions regarding male partners involvement, experience and opinion on PMTCT. [file 8617540.f1.docx]

## Annex: Questionnaires

QUESTIONNAIRES –ENGLISH VERSION

JIMMA UNIVERSITY, COLLEGE HEALTH SCIENCES

DEPARTMENT OF EPIDEMIOLOGY

Informed consent form

My name is ____________________________________________; I am working as a data collector temporarily for post graduate student of Jimma University College of Public health &Medical Science. This study will be conducted with objective of assessing male partner involvement in PMTCT of HIV/AIDS and associated factors among male reproductive age in Lemo Woreda, Hadiya Zone, and South Ethiopia. The study is directly related to male partners of pregnant women who attend ANC service in the public health care facilities six month prior this study conducted. You are selected to participate in this study just by chance so your participation in the study is upon purely voluntary basis .Your willingness and cooperation for the interview is helpful in identifying problems related to male partners involvement in PMTCT of HIV/AIDS and will give us is quite useful information to achieve the objective of the study.

I would like to interview you a few questions about your experience and opinion of PMTCT services with your woman’s/partner’s . It will take about 20 - 30 minutes. The information you provide to us is completely confidential and will not be shared with anyone else without your consent. Your name or any identifying information will not be registered. You may refuse to answer any question and choose to stop the interview at any time. If you agree to participate in this study I will interview you.

Would you be willing to participate? Yes No

If the answer is yes, thanks! Conduct the interview. If the answer is no, Thanks! Proceed to the next eligible client.

Interviewer name**: _______________________** and Signature **____________**

Date**: __/__/____**started Time _____ finished Time __________

Name of Kebele: ______________________House code: __________

Name of supervisory __________and Signature _____________________

**Section I: Socia-Demograpic and Socio-Economic Characteristics**

**Instruction**: - please, circle in your appropriate response to the following socio-demographic questions

| S./No. | Questions | Response category | Skip |
| --- | --- | --- | --- |
| 101 | What is your age? | …….years |  |
| 102 | Residence of the respondent | 1. urban  2. rural |  |
| 103 | What is your ethnicity? | 1. Hadiya 2. Kamibata 3. Amhara 4. Gurage 5. Silte 6. Others (specify)………… |  |
| 104 | What is your religion? | 1. Protestant 2. Orthodox 3. Muslim 4. Catholic 5. Adventist 6. Others (specify)………….. |  |
| 105 | What is your Marital status? | 1. Traditional marriage 2. Registered marriage 3. Cohabitating 4. separated/divorced 5. Other(specify)……………… |  |
| 106 | For how long have you been living with the current wife/partner? (together cohabitating or in marriage) | ………. Years |  |
| 107 | Are you currently living with your partner in the same house? | 1. Yes 2. No 3. Don’t know |  |
| 108 | What is the highest level of education you completed? | 1. Illiterate 2. Able to read and write 3. Grade 1 – 4 4. Grade 5 – 8 5. Grade 9–10 6. Grade 11 and higher 7. Others (specify)---------- |  |
| 109 | What is your occupation? | 1. Farmer 2. Government Employee 3. Merchant 4. Daily laborer 5. Other (specify)………. |  |
| 1010 | Your estimated family income in year  Wheat--------------in konital  Teff----------------- in konital  Barley--------------in konital  In cash--------------ETB  Others (specify)---------------- | --------------ETB (average family monthly income) |  |

**Section II:-Socio-cultural factors of male partners about PMTCT of HIV/AIDS.**

**Instruction**: - For the following statements related to socio-cultural factor of male partner about PMTCT of HIV//AIDS, please express your opinion by stating “strongly agree”, “agree”, “uncertain”, “disagree” or “strongly disagree” .

**Key**:SA- Strongly agree ,A- Agree ,U- Undecided, D- Disagree, SD­­­- Strongly disagree

| S/No | Respondents opinions on socio-cultural factors | Scales of agreement of the respondents | | | | |
| --- | --- | --- | --- | --- | --- | --- |
|  |  | SA | A | U | D | SD |
| 201 | Men should accompany their pregnant wives to  ANC/PMTCT | 5 | 4 | 3 | 2 | 1 |
| 202 | A pregnant woman can be tested for HIV even if her partner disagrees. | 5 | 4 | 3 | 2 | 1 |
| 203 | It is a taboo for men to discuss with women about HIV  testing During pregnancy, delivery and breastfeeding | 1 | 2 | 3 | 4 | 5 |
| 204 | Even If couples believe they are faithful to each other, they should be tested for HIV together during ANC follow up for the sake of PMTCT. | 5 | 4 | 3 | 2 | 1 |
| 205 | It suffices/enough that a pregnant woman be accompanied to ANC clinic by less busy relatives/family members | 1 | 2 | 3 | 4 | 5 |
| 206 | An HIV test result of a pregnant woman indirectly confirms HIV status of her partner. | 1 | 2 | 3 | 4 | 5 |
| 207 | If a pregnant woman found to be HIV positive she  should be divorced | 1 | 2 | 3 | 4 | 5 |
| 208 | Couples can use condoms to reduce chances of  mother to child transmission | 5 | 4 | 3 | 2 | 1 |
| 209 | It is better to live with unknown HIV status than live depressed with positive HIV status known. | 1 | 2 | 3 | 4 | 5 |
| 2010 | A positive HIV test result of a female partner proves that she is unfaithful? | 1 | 2 | 3 | 4 | 5 |
| 2011 | ANC/PMTCT clinics are give services for women and children only ⃰ | 1 | 2 | 3 | 4 | 5 |
| 2012 | It is better to postpone HIV testing to post delivery as pregnancy by itself is stressful | 1 | 2 | 34 | 5 | 6 |
|  | Total Score |  |  |  |  |  |

**Section II: Knowledge of male partners on PMTCT of HIV/AIDS**

**Instruction**: For the statements assessing knowledge and awareness of male partner on HIV/PMTCT, please, answer “yes” if you agree, “No” if you disagree or “don’t know” if you are uncertain.

| S/No | Questions to male partner | | | Response category | Skip |
| --- | --- | --- | --- | --- | --- |
| 301 | HIV is transmitted through? | | | |  |
| 301.01 | Unprotected Sexual intercourse | 1. Yes 2. No. 3. Don’t know | | |  |
| 301.02 | Eating from same plate | 1. Yes 2. No. 3. Don’t know | | |  |
| 301.03 | Contaminated sharps/needles | 1. Yes 2. No. 3. Don’t know | | |  |
| 301.04 | Blood transfusion | 1. Yes 2. No. 3. Don’t know | | |  |
| ***302*** | Mother- to-child transmission of HIV | | | |  |
| 302.01 | HIV can be transmitted from mother to child | 1. Yes 2. No. 3. Don’t know | | |  |
| 302.02 | HIV can be transmitted from infected mother to child during pregnancy | 1. Yes 2. No. 3. Don’t know | | |  |
| 302.03 | HIV can be transmitted from infected mother to child during labor and delivery | 1. Yes 2. No. 3. Don’t know | | |  |
| 302.04 | HIV can be transmitted from infected mother to child while sleeping with baby on same bed | 1. Yes 2. No. 3. Don’t know | | |  |
| 302 .05 | HIV can be transmitted from infected mother to child during breast feeding after birth | 1. Yes 2. No. 3. Don’t know | | |  |
| ***303*** | What helps to reduce mother to child transmission of HIV***?*** | | | |  |
| 303.01 | HIV counseling and testing for pregnant mothers | | 1. Yes 2. No. 3. Don’t know | |  |
| 303.02 | HIV counseling and testing for male partners | | 1. Yes 2. No. 3. Don’t know | |  |
| 303.03 | Antiretroviral drugs to infected mother and baby born from her | | 1. Yes 2. No. 3. Don’t know | |  |
| 303.04 | Delivery by cesarean section | | 1. Yes 2. No. 3. Don’t know | |  |
| 303.05 | Complete avoidance of breast feeding is one option | | 1. Yes 2. No. 3. Don’t know | |  |
| 303.06 | Exclusive breast feeding for first 6 months is one option | | 1. Yes 2. No. 3. Don’t know | |  |
| 303.07 | Using contraception by HIV positive couple | | 1. Yes 2. No. 3. Don’t know | |  |
| 304 | Have you ever heard about a program called Prevention of Mother-To-Child Transmission (PMTCT) of HIV? | | 1. Yes 2. No. 3. Don’t know | |  |
| 305 | Do you know that PMTCT services are offered in all government health facilities? | | 1. Yes 2. No. 3. Don’t know | |  |
| *306* | Do you know that pregnant women are counseled and tested at antenatal care clinic? | | 1. Yes 2. No. 3. Don’t know | |  |

**Section IV: Male partner’s experience and opinion regarding PMTCT service related factors.**

**Instruction**:- For the following statements related to PMTCT of HIV/AIDS programmatic factors, please express your opinion by stating “strongly agree”, “agree”, “undetermined”, “disagree” or “strongly disagree”.

**Key**: SA- Strongly agree, A- Agree, U- Undecided, D- Disagree,

SD­­­- Strongly disagree

| S/NO | Respondents opinions socio-cultural factors | Scales of agreement of the respondents | | | | |
| --- | --- | --- | --- | --- | --- | --- |
|  |  | SA | A | U | D | SD |
| 401 | Antenatal clinics should be opened on weekends and evening for men to attend the ANC clinics with their partner. | 1 | 2 | 3 | 4 | 5 |
| 402 | Distance from health facility was major obstacle for you to attend ANC/PMTCT clinic with your partner. | 1 | 2 | 3 | 4 | 5 |
| 403 | Couple HIV counseling and testing for PMTCT should be conducted at villages | 5 | 4 | 3 | 2 | 1 |
| 404 | There should be separate waiting areas for men and women visiting maternal and child health clinics | 1 | 2 | 3 | 4 | 5 |
| 405 | There should be a different exit after HIV testing to avoid being identified by the crowd waiting for service | 1 | 2 | 3 | 4 | 5 |
| 406 | From what you have observed or heard service providers don’t request men in waiting area to enter in to ANC together with their partner | 1 | 2 | 3 | 4 | 5 |
| 407 | From what you have observed or heard health facilities do give men medical certificate of ANC attendance | 5 | 4 | 3 | 2 | 1 |
| 408 | As you have noticed health facilities inviting/promoting  male participation in PMTCT through Mass media | 5 | 4 | 3 | 2 | 1 |
| 409 | As you have ever seen a sign board with picture or message promoting male participation in PMTCT at gate or in premise of any health facilities | 5 | 4 | 3 | 2 | 1 |
| 4010 | If your partner had ANC follow up, have you been invited verbally or in written for your attendance by the antenatal clinic | 5 | 4 | 3 | 2 | 1 |

**Section VI. Level of male partner involvement in PMTCT of HIV/AIDS**

**Instruction**:-for the questions assessing male partner involvement in PMTCT of HIV/AIDS, please answer “yes” if you are agree , “No” if you are disagree or “don’t know” if you are uncertain

| S/No | Questions to male partner | Response category | Skip |
| --- | --- | --- | --- |
| 501 | Have you ever self initiated the discussion on importance of PMTCT service with your partner during this pregnancy? | 1. Yes 2. No. 3. Don’t know |  |
| 502 | Have you ever requested your wife to be tested for HIV during this pregnancy? | 1. Yes 2. No. 3. Don’t know |  |
| 503 | If your partner had ANC follow up, have you ever asked her what information/service she got at ANC clinic | 1. Yes 2. No. 3. Don’t know  4. NA (my partner had no ANC follow up) | If 4  Go to Q. 8 |
| 504 | Have you ever reminded your partner of her ANC follow up (schedule)? | 1. Yes 2. No. 3. Don’t know |  |
| 505 | Did you cover medical expenses of your partner in the ANC follow up of this pregnancy? | 1. Yes 2. No. 3. Don’t know |  |
| 506 | Did you accompany her to ANC clinic at least once during this pregnancy? | 1. Yes 2. No. 3. Don’t know | If 2 go to Q.8 |
| 507 | If yes to Q.6 did you enter in to ANC room together with your partner? | 1. Yes 2. No. 3. Don’t |  |
| 508 | Were you counseled and tested for HIV during your spouse’s/partner’s pregnancy? | 1. Yes 2. No. 3. Don’t know 4. I tested HIV+ pre-pregnancy | If 2 go to10 |

| 509 | If yes to Q 8, were you counseled and tested together with your partner? | 1. Yes 2. No. 3. Don’t know 4. NA (she is already positive) |  |
| --- | --- | --- | --- |
| 5010 | Assuming you are willing for HIV test now, will you confide in your female partner if you test positive for HIV? | 1. Yes 2. No. 3. Don’t know 4.NA (he already positive ) |  |
| 5011 | Assuming your female partner gives consent for HIV test and tests positive, will you accept that she and the newborn take ARVs for PMTCT | 1. Yes 2. No. 3. Don’t know 4.NA (she is already positive) |  |
| 5012 | Assuming your female partner tests HIV positive, would you be confident to help in the newborn’s medical follow up until the HIV status is known? | 1. Yes 2. No. 3. Don’t know 4.NA (she already positive) |  |
| 5013 | Assuming your female partner and you both gives consent for HIV test and your female partner tests positive while your test negative, would you decide to discontinue your conjugal or love relationship | 1. Yes 2. No. 3. Don’t know 4. NA(she is already positive) |  |
| 5014 | Assuming you and your partner are HIV tested, If you are positive while she is negative, would you be confident to use condom consistently to prevent transmission to her and thus to the child? | 1. Yes 2. No. 3. Don’t know |  |

THANK YOU!!!!!!!!!!!!!!
